# Depression with risks for spontaneous abortion: a meta-analysis

**DOI:** 10.1186/s40359-025-03484-4

**Published:** 2025-10-14

**Authors:** Junxiu Liu, Mingyang Zhao, Jia Zhuan, Yanmin Song, Zhe Han, Yuanyuan Zhao, Hua Ma, Xiumei Yang

**Affiliations:** Department of Gynecology, Cangzhou People’s Hospital, 7 Qingchi Avenue, Xinhua District, Cangzhou City, Hebei Province China

**Keywords:** Depression, Risks, Spontaneous abortion, Meta-analysis

## Abstract

**Background:**

To assess the association between depression in women and the risk of spontaneous abortion (SA) after pregnancy.

**Methods:**

Relevant studies were identified through searches of the PubMed, Web of Science, and Embase databases. The pooled effect sizes were presented as relative risk (RR) along with their respective 95% confidence intervals (95% CI), and data analysis was conducted using the random-effects model.

**Results:**

A total of 31 studies involving 8,919,953 women were included in this meta-analysis. The results indicated a significant association between depression and increased risk of SA (RR = 1.34, 95% CI [1.27, 1.41], *p* < 0.001; I^2^ = 87%). Sensitivity analysis did not identify outlier studies. Subgroup analysis enhanced the robustness and credibility of the results. Egger regression test revealed a low risk of bias.

**Conclusions:**

Compared to women without depression, women with depression is associated with an increased risk of SA after pregnancy, an association that is unrelated to the use of antidepressant medication.

**Supplementary Information:**

The online version contains supplementary material available at 10.1186/s40359-025-03484-4.

## Introduction

As the largest single contributor to the global burden of disease, currently over 264 million people are affected by depression. Women (5.1%) are more likely to suffer from depression than men (3.6%) [1]. The prevalence of antenatal depression among childbearing women is as high as 28% [2]. As another public health issue, spontaneous abortion (SA) in pregnant women is also gradually receiving more attention from society. The latest research shows that due to factors such as increasing maternal age, increasing age of partners, changes in body mass index, smoking, alcohol consumption, stress, air pollution, and other various risk factors, the rate of SA in women can reach as high as 15.3% [3]. Compared to women without a history of SA or those experiencing their first pregnancy, women with a history of SA are more likely to develop depression [4]. Therefore, SA not only affects the body and economic life, but is also a high-risk factor for depression. However, the potential role of depression itself as a risk factor for SA remains underexplored. Although several meta-analyses have examined the association between antidepressant use and SA, their findings are inconsistent, with some reporting an increased risk [1, 5, 6], and others showing no significant association [7–9]. Similarly, observational studies investigating depression independent of medication have also yielded conflicting conclusions [10–12]. The biological and behavioral mechanisms plausibly linking depression to SA further underscore the need for clarity on this relationship: Biologically, depression is associated with dysregulation of the hypothalamic-pituitary-adrenal (HPA) axis, leading to elevated cortisol levels [13]. Behaviorally, depression can lead to poor nutrition, sleep disturbances, substance use, and reduced adherence to prenatal care, all of which are potential risk factors for adverse pregnancy outcomes [14]. Nevertheless, to our knowledge, a preliminary search of PROSPERO and Cochrane Library revealed no existing or ongoing systematic reviews and meta-analyses that specifically investigate the association between depression itself and the risk of SA, distinguishing it from the effects of antidepressant medication.

Therefore, our aim is to conduct a meta-analysis on the relationship between women with depression and the risk of SA, with the hope of providing some useful management guidance for clinical physicians.

## Materials and methods

### Search strategy

Studies related to depression and SA were searched in the PubMed, Web of Science, and Embase databases. The following terms were used for depression searches: “depression”, “depressive symptom”, “psychiatric disorders”, “antidepressant”, “selective serotonin-reuptake inhibitors”, “SSRIs”, “citalopram”, “fluoxetine”, “fluvoxamine”, “paroxetine”, “sertraline”, “dual action agents”, “DAAs”, “nefazodone”, “trazodone”, “venlafaxine”, “tricyclics”, “TCAs”, “amitriptyline”, “amoxapine”. “clomipramine”, “desipramine”, “doxepin”, “imipramine”, “maprotiline”, “nortriptyline”, “protriptyline”, “trimipramine”, “monoamine oxidase inhibitors”, “moclobemide”, “phenelzine”, “tranylcypromine”. Search terms for SA included: “SA”, “miscarriage”, “early pregnancy loss”, “Tubal Abortion”. We additionally reviewed the reference lists of included studies and relevant review articles to identify any potentially eligible studies that might not have been captured by our electronic database searches.

### Inclusion and exclusion criteria

Studies meeting the following criteria were included: (1) The study report should be written in English; (2) The study was a cohort (prospective or retrospective) or case-control design; (3) The study population included women with or without depression diagnosed at baseline, prior to any observed outcome. For the purpose of this meta-analysis, ‘depression itself’ refers to a diagnosis of a depressive disorder (based on medical records, clinical assessment, or diagnostic scales) or a state of depression, regardless of treatment with antidepressants. The non-depressed group mainly includes healthy women or women who have not been exposed to antidepressants; (4) The outcome was incident SA, including both sporadic SA and recurrent spontaneous abortion (RSA); (5) Sufficient data were provided to estimate the effect size and 95% confidence intervals (CIs), including risk ratios (RR), hazard ratios (HR), prevalence ratios (PR), and odds ratios (OR). Exclusion criteria were as follows: (1) Cross-sectional studies, reviews, comments, conference abstracts, or case reports; (2) Studies that enrolled women with a history of SA at baseline; (3) Studies where depression was assessed after the SA event; (4) Studies without a non-depressed comparison group; (5) Duplicate publications or overlapping datasets.

### Data extraction and quality assessment

Two researchers (J.L. and M.Z.) independently reviewed the titles and abstracts of these articles and selected studies that met the inclusion criteria for full-text review. Reviews, animal studies, irrelevant studies or conference studies were excluded at this stage. The full texts of the remaining articles were then assessed for eligibility. For studies with the same population resources or overlapping datasets, the most comprehensive study was included. Details and data were independently extracted by the two researchers (J.Z. and Y.S.) in a standardized electronic spreadsheet, with discrepancies resolved by a third researcher (X.Y.) until consensus was reached for each study. Information extracted from each study includes: First author, year; Study period; Geographic region; Study design; Diagnosis of depression; Definition of SA; Depressed group; Non-depressed group; Sample size; Adjustment for confounding factors; Adjusted (when available) or unadjusted RR, HR, PR, OR, and 95% CIs. The Joanna Briggs Institute (JBI) Critical Appraisal Tool was used for the assessment of study quality [15]. The range of scores for the quality assessment of included studies is from 0 to 8 points, with 8 points representing the highest quality level of a study. The results of the critical appraisal were incorporated into the synthesis by conducting a subgroup analysis based on JBI scores to assess the potential impact of study quality on the pooled effect size.

### Statistical analyses

All analyses were conducted in observational studies, including cohort studies and case-control studies. RR, HR, OR, and PR were used as effect size to assess the relationship between depression and SA. If covariate adjustment was performed in the included studies, we used the adjusted data. Alternatively, we used unadjusted data. The effect size and their corresponding 95% CIs data from each study were extracted and summarized. Heterogeneity of the effect across studies was assessed by Q statistics. I^2^ statistics were provided to quantify the percentage of total variation across studies that was attributable to heterogeneity rather than to chance. An I^2^ value exceeding 50% indicated significant diversity in findings. Sensitivity analyses were conducted to identify potential outlier studies. Subgroup analyses were conducted based on different geographic regions, different study designs, different definitions of SA, depressed and non-depressed groups, Whether adjusting confounding factors, and different JBI Score.

We conducted a visual inspection of the funnel plot to assess for publication bias, where asymmetry indicates the presence of publication bias and symmetry indicates its absence. The Egger test was employed to quantify funnel plot asymmetry, with a significance level set at *P*<0.05. All statistical analyses were conducted using STATA 14.0.

## Results

### Literature search

**Fig. 1:**
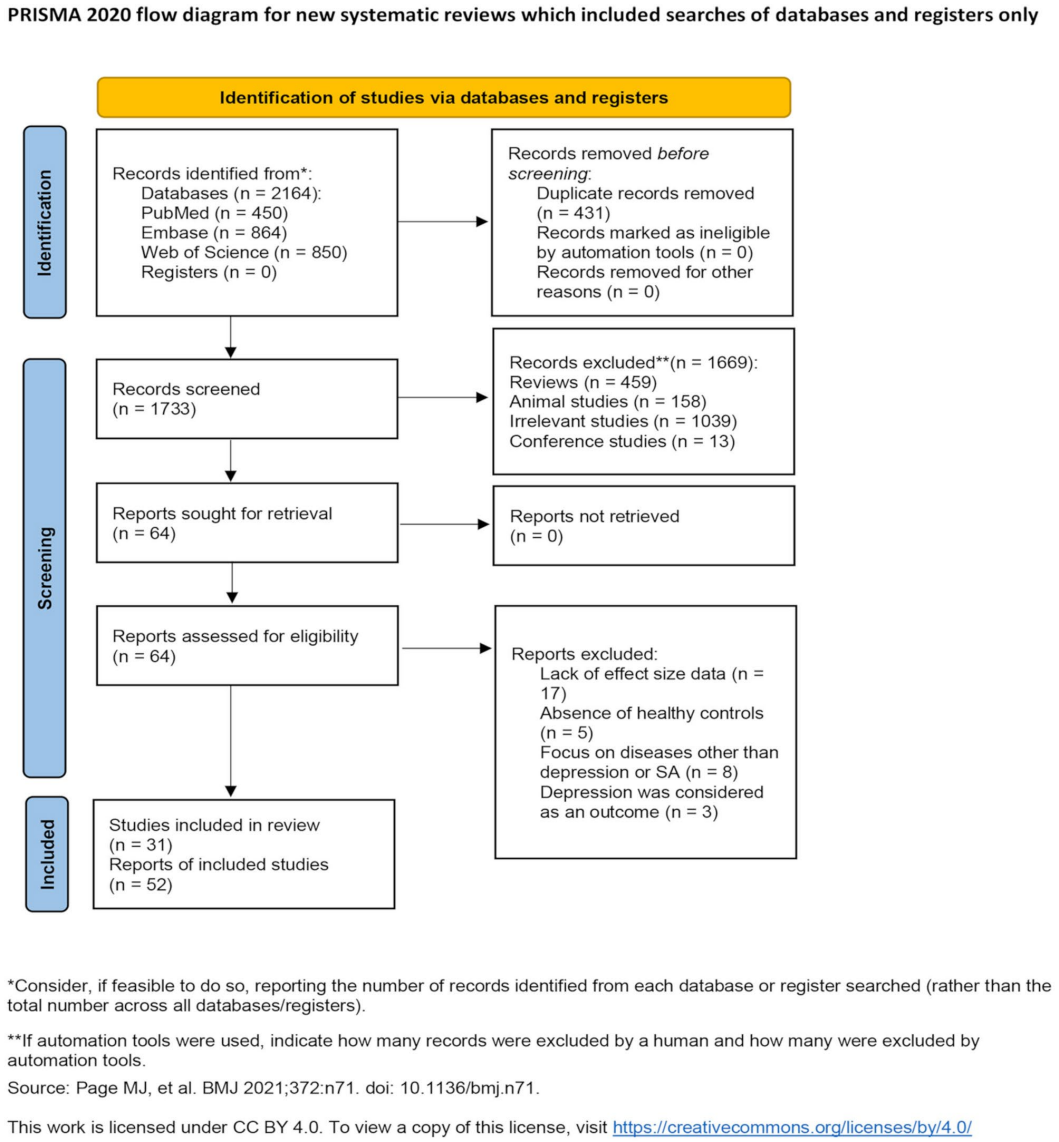
Flow chart of search and study identifcation

Through the initial search, we identified 2164 studies (450 in PubMed, 864 in Embase, 850 in Web of Science). After removing duplicates, 1733 studies remained. Further exclusion based on titles and abstracts was conducted, including reviews (*n* = 459), animal studies (*n* = 158), irrelevant studies (*n* = 1039), and conference studies (*n* = 13). The remaining 64 studies underwent full-text evaluation. Of these 64 studies, 33 were excluded due to lack of effect size data (*n* = 17), absence of healthy controls (*n* = 5), focus on diseases other than depression or SA (*n* = 8), or studies where depression was considered as an outcome (*n* = 3). Finally, 31 studies were included (Fig. 1) [10–12, 16–43].

### Study characteristics and quality assessment

All 31 studies, published between 1993 and 2024, included a total of 8,919,953 women (816,407 with depression and 8,103,546 without depression). These studies involved populations from diverse geographic regions: Australia (1 study), North America (5 studies), Europe (12 studies), non-European countries (8 studies), Asia (1 study), and multiple countries worldwide (4 studies). Among the four multinational studies, which utilized international databases or collaborative cohorts, populations were primarily drawn from Canada, Switzerland, and Italy, in addition to other global region. Twenty-three studies were prospective cohort studies, three were retrospective cohort studies, and five were case-control studies. Definitions of SA: Five studies reported SA before 20 weeks, five studies before 22 weeks, one study before 24 weeks, three studies reported RSA, and eighteen studies did not specify the timing. Definitions of the depression group: Four studies diagnosed individuals as depressed, taking antidepressants or not. Twenty-three studies involved individuals taking antidepressants, while four studies reported cases of depression without mentioning antidepressants use. Definition of the non-depression group: Twenty studies classified individuals as healthy, and eleven studies included individuals who had never been exposed to antidepressants. Twenty studies adjusted for confounding factors, while eleven studies did not (Table 1). The quality of all included studies was evaluated using the JBI. Overall, the quality of the included studies was high, with ten studies scoring 6 points and twenty-one studies scoring 8 points (Additional Table 1).

**Table 1:** Characteristics of the included studies

First author, Year	Study period	Geographic region	Study design	Diagnosis of depression	Definition of SA	Depressed group	Non-depressed group	Sample size		Adjustment for confounding factors
								Depressed group	Non-depressed group	
Liang, 2004	1996–2018	Australia	Prospective cohort	Medical record	< 20 weeks, RSA	Depressed, taking or not taking antidepressants	Healthy	3,136	5,571	Yes
Almeida, 2016	1998–2002	Canada	Retrospective cohort	Medical record	< 20 weeks	Depressed, taking or not taking antidepressants	Healthy	12,674	28,343	Yes
Ban, 2016	1990–2009	UK	Prospective cohort	Medical record	NR	Depressed, taking or not taking antidepressants	Healthy	121,909	390,665	Yes
Yaris, 2005	1999–2004	Non-European countries	Case-control	Medication record	NR	Taking antidepressants	Healthy	124	248	No
Einarson, 2009	NR	Non-European countries	Prospective cohort	Medication record	NR	Taking antidepressants	Healthy	937	937	Yes
Richardson, 2019	1995–2018	UK	Prospective cohort	Medication record	< 24 weeks	Taking antidepressants	Healthy	281	1,045	Yes
Ankarfeldt, 2021	2004–2016	Denmark	Prospective cohort	Medication record	NR	Taking antidepressants	Never been exposed to antidepressants	1,212	1,018,745	Yes
Sjaarda, 2021	2007–2011	USA	Prospective cohort	Medication record	NR	Taking antidepressants	Never been exposed to antidepressants	97	674	No
Kitchin, 2022	2002–2016	Spanish	Case-control	Medication record	< 22 weeks	Taking antidepressants	Never been exposed to antidepressants	9,621	62,658	Yes
Wu, 2019	2000–2012	USA	Prospective cohort	Medication record	< 20 weeks	Taking antidepressants	Never been exposed to antidepressants	223	5,228	Yes
Andersen, 2014	1997–2010	Denmark	Prospective cohort	Medication record	NR	Taking antidepressants	Never been exposed to antidepressants	22,884	1,256,956	Yes
Nakhai-Pour, 2010	1998–2003	USA, Canada	Case-control	Medication record	NR	Depressed, taking or not taking antidepressants	Never been exposed to antidepressants	2,777	53,587	Yes
Kolding, 2021	2007–2014	Denmark	Prospective cohort	Medication record	< 22 weeks	Taking antidepressants	Never been exposed to antidepressants	4,105	353,581	Yes
Ostenfeld, 2022	1997–2016	Denmark	Prospective cohort	Medication record	< 22 weeks	Taking antidepressants	Never been exposed to antidepressants	1,900	7,600	Yes
Evans-Hoeker, 2018	2009–2012	Non-European countries	Prospective cohort	PHQ-9 score	NR	Taking antidepressants	Never been exposed to antidepressants	90	1,484	Yes
Johansen, 2014	1996–2002	Denmark	Prospective cohort	Medication record	< 22 weeks	Taking antidepressants	Never been exposed to antidepressants	26,040	1,165,124	Yes
Kjaersgaard, 2013	1997–2018	Denmark	Prospective cohort	Medication record	< 22 weeks	Taking antidepressants	Never been exposed to antidepressants	22,317	998,645	Yes
Klieger-Grossmann, 2013	NR	Multiple countries	Prospective cohort	Medication record	NR	Taking antidepressants	Healthy	213	212	No
Wang, 2021	2017–2019	China	Case-control	SDS	RSA	Depression without mention of antidepressants use	Healthy	1,139	1,419	Yes
Sivojelezova, 2005	1999–2000	Non-European countries	Prospective cohort	Medication record	NR	Taking antidepressants	Healthy	132	132	No
Chun-Fai-Chan, 2005	NR	Multiple countries	Prospective cohort	Medication record	NR	Taking antidepressants	Healthy	91	89	No
Einarson, 2003	NR	Multiple countries	Prospective cohort	Medication record	NR	Taking antidepressants	Healthy	147	147	No
Einarson, 2001	NR	Multiple countries	Prospective cohort	Medication record	NR	Taking antidepressants	Healthy	150	150	No
kulin, 1998	NR	Non-European countries	Prospective cohort	Medication record	NR	Taking antidepressants	Healthy	267	267	No
Goldstein, 1997	-1996	Non-European countries	Prospective cohort	Medication record	NR	Taking antidepressants	Healthy	759	3,216	No
Koren, 1996	NR	Non-European countries	Prospective cohort	Medication record	NR	Taking antidepressants	Healthy	128	128	No
Pastuszak, 1993	NR	Non-European countries	Prospective cohort	Medication record	NR	Taking antidepressants	Healthy	128	128	No
Hope, 2022	1990–2017	UK	Retrospective cohort	Medical record	RSA	Depression without mention of antidepressants use	Healthy	510,490	2,150,306	Yes
Diav-Citrin, 2008	1994–2005	Israel, Italy, Germany	Prospective cohort	Medication record	NR	Taking antidepressants	Healthy	710	1,467	Yes
Magnus, 2021	2010–2016	Norway	Case-control	Medical record	< 20 weeks	Depression without mention of antidepressants use	Healthy	71,551	593,009	Yes
Gold, 2007	1990-	USA	Retrospective cohort	Medical record	< 20 weeks	Depression without mention of antidepressants use	Healthy	175	1,785	Yes

Abbreviations: NR, not reported; UK, United Kingdom; USA, the United States of America; PHQ-9, patient health questionnaire; SDS, self-rating depression scale; CESD-10, 10-item Center for Epidemiologic Studies Depression Scale; SA, spontaneous abortion; RSA, recurrent spontaneous abortion

### Synthesis of results

#### The association between depression and SA

**Fig. 2:**
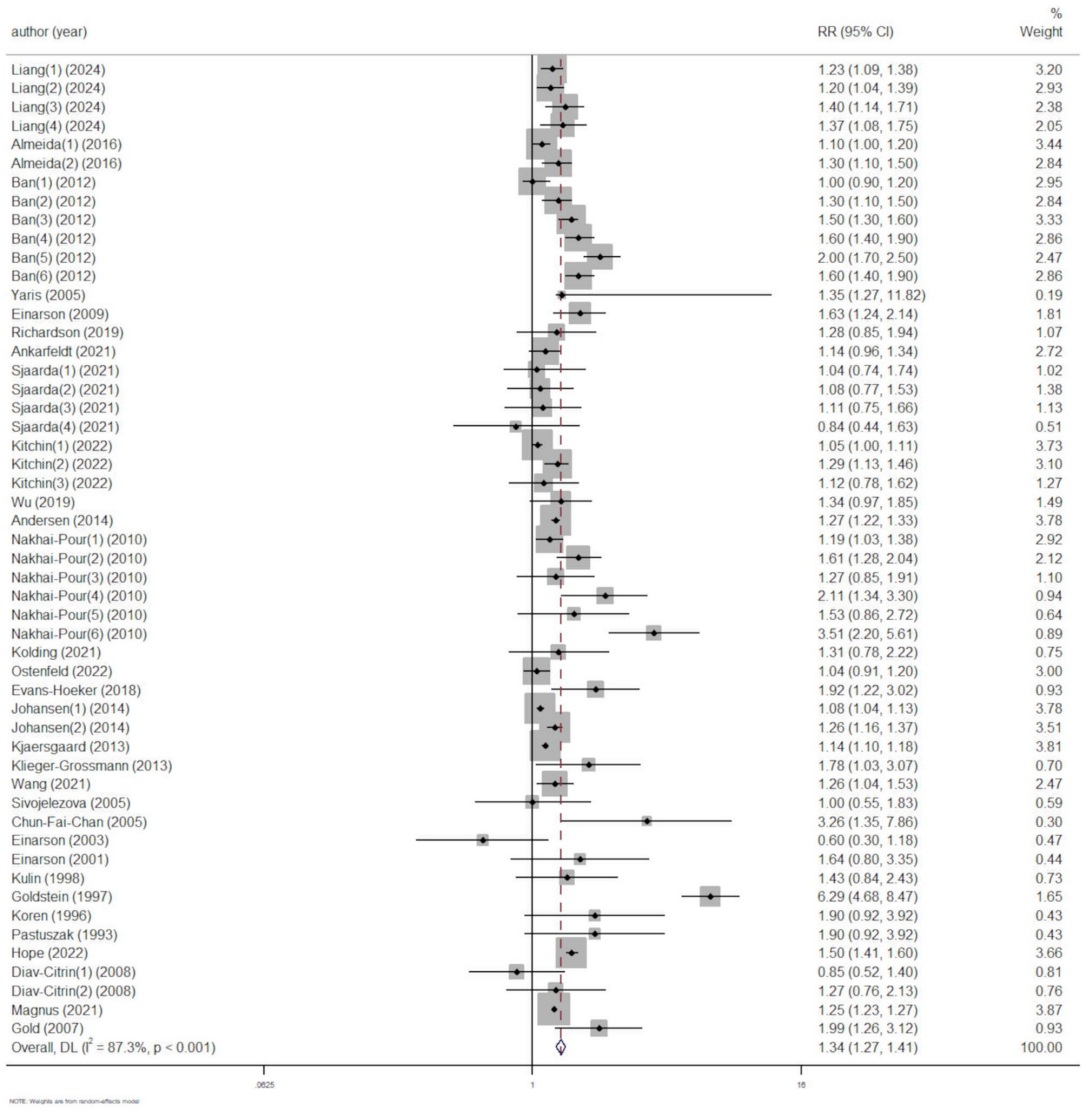
Forest plot of the association between depression and risk of SA

We conducted a meta-analysis using a random-effects model on all 31 studies (comprising 52 comparisons). The results indicated a significant association between depression and increased risk of SA (RR = 1.34, 95% CI [1.27, 1.41], *p* < 0.001; I^2^ = 87%) (Fig. 2). Sensitivity analysis revealed no outlier studies (Additional Fig. 1).

#### Subgroup analyses

To further explore the impact of different populations and study characteristics on the results, we conducted subgroup analysis. Geographic regions included Europe, North America, Non-European countries, multiple countries, Australia, and Asia. Study designs included prospective cohort study, retrospective cohort study, and case-control study. Definitions of SA included < 20 weeks, < 22 weeks, < 24 weeks, RSA and not reported. The depression group included three subgroups: (1) depression without mention of antidepressants use, (2) depression without taking antidepressants, and (3) taking antidepressants. The non-depression group includes two subgroups: (1) healthy women and (2) women who have never been exposed to antidepressants. Adjustment for confounding factors was defined as yes or no. All studies had JBI scores of 6 or 8. Firstly, among women including Europe, North America, Asia, Australia, and non-European regions, depression was found to be associated with an increased risk of SA. However, this association was not found in the subgroup analysis of studies involving multiple countries worldwide. Secondly, a significant positive association between depression and SA was found in both prospective cohort studies, retrospective cohort studies, and case-control studies. Thirdly, in subgroups with different definitions of SA, depression was found to be associated with an increased risk of SA, except in the subgroup under 24 weeks. Fourthly, in each subgroup defined by depression and non-depression, we found a significant positive association between depression and SA. Fifthly, this significant positive association was found in the subgroup adjusted for confounding factors rather than in the subgroup not adjusted for confounding factors; Sixthly, this significant positive association exists in subgroups with different JBI Score (Additional Table 2).

#### Publication bias

**Fig. 3:**
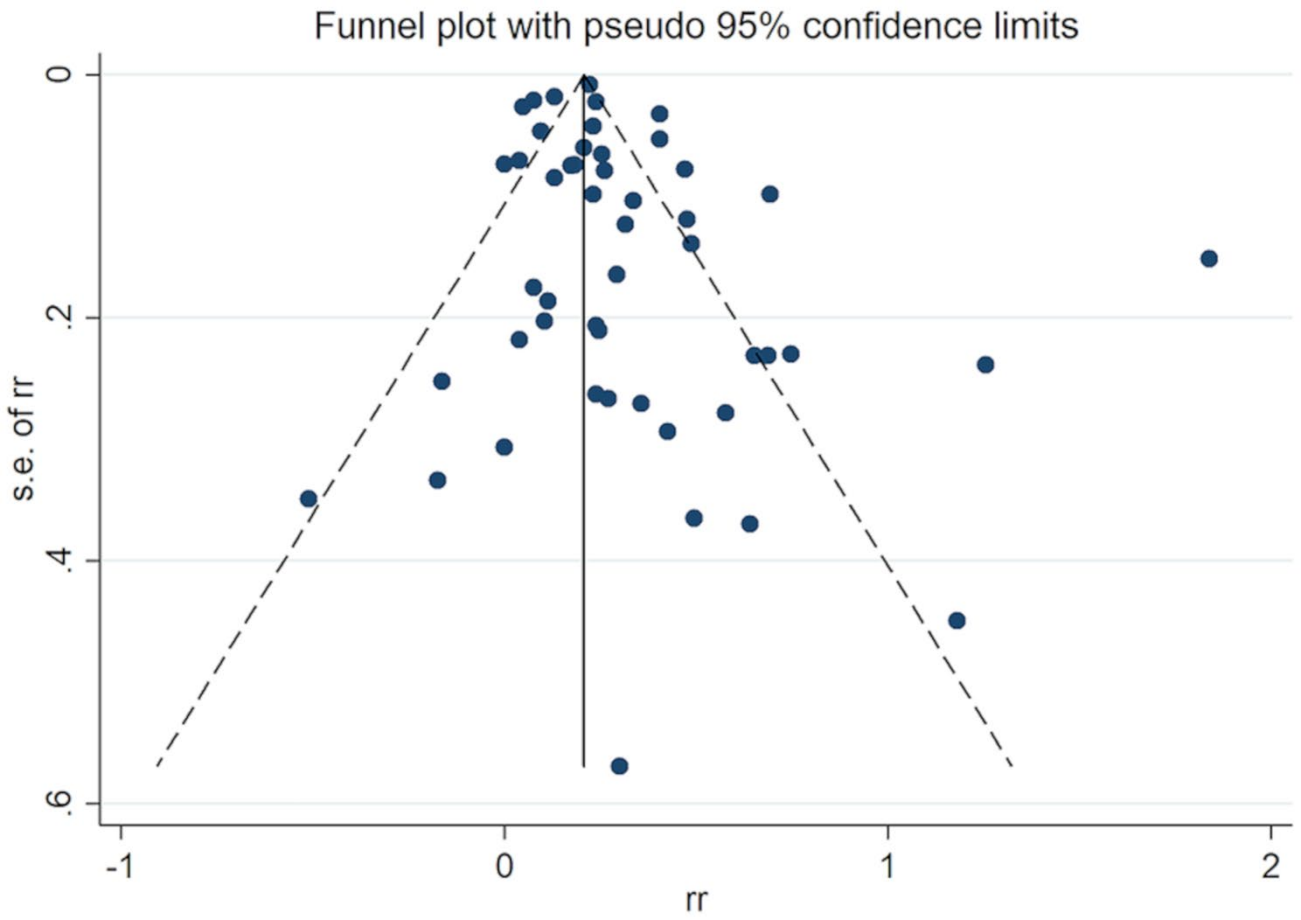
The funnel plot

**Fig. 4:**
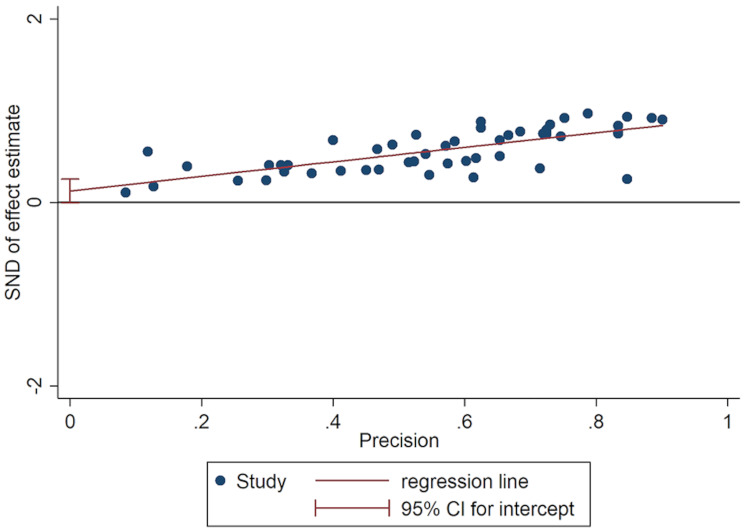
The Egger regression test

The distribution of studies in the funnel plot is generally symmetrical, but individual studies may exhibit publication bias (Fig. 3). Therefore, we further conducted the Egger regression test and found a low risk of bias (*P* = 0.055) (Fig. 4).

## Discussion

This meta-analysis indicates that women with depression is significantly associated with an increased risk of SA. Several meta-analyses have previously explored the relationship between women’s use of antidepressants and SA, but the conclusions have not been consistent [1, 5–9]. The reasons may be related to factors such as the types of antidepressants, the timing of antidepressants use before or during pregnancy, statistical methods, and the inclusion of different types of studies. It is worth noting that as of now, there is no meta-analysis investigating the relationship between depression itself, rather than antidepressants, and the risk of SA. To our knowledge, this study is the first comprehensive meta-analysis evaluating the relationship between depression and the risk of SA.

The biological mechanisms underlying the association between depression and increased risk of SA remain incompletely understood and are primarily supported by clinical observations rather than direct animal or experimental evidence. The strongest evidence points to dysregulation of the HPA axis [13]. During pregnancy, elevated estrogen levels double corticosteroid-binding globulin, prolonging plasma cortisol half-life [44]. In women with depression, HPA axis hyperactivity leads to excessive cortisol production, potentially exacerbating already elevated cortisol levels during pregnancy. This may adversely affect placental development and the intrauterine environment, thereby increasing SA risk [13, 45]. Other plausible pathways, though supported by less direct evidence, include shared neuroendocrine pathways between the brain and placenta. Both tissues synthesize peptides and proteins such as brain-derived neurotrophic factor, oxytocin, vascular endothelial growth factor, cortisol, and matrix metalloproteinases. Whether the interaction between the neurochemical mechanism and the placental mechanism could mediate the occurrence of adverse obstetric outcomes such as SA in women with mental disorders is worthy of further exploration [46]. Additional mechanisms may involve depression-related suppression of the hypothalamic–pituitary–gonadal and hypothalamic–pituitary–thyroid axes, leading to decreased estradiol and reduced thyroid hormone levels—both established risk factors for SA [47–49]. Furthermore, depression-associated behavioral changes and clinical manifestations—such as hyperemesis gravidarum, sexual dysfunction, weight changes, smoking, and alcohol use—may represent another pathway contributing to increased SA risk [50–53]. It is important to note that most of these mechanisms are derived from indirect clinical associations and pathophysiological reasoning. To date, no dedicated animal models or prospective clinical studies have conclusively established causality between depression and SA via these pathways, highlighting an important area for future investigation.

Due to the high heterogeneity of the results, subgroup analyses were conducted to determine several study-level variables that may lead to heterogeneity, including different geographic regions, different study designs, different definitions of SA, depressed and non-depressed groups, Whether adjusting confounding factors, and different JBI Score. Firstly, the finding that depression was associated with an increased risk of SA has been confirmed in various regions such as Europe, Asia, North America, and Australia. However, this association was not found in subgroup analysis of studies including multiple countries around the world, possibly due to small sample sizes and the lack of adjustment for confounding factors in these studies, which may lead to significant bias to the results. Secondly, through subgroup analyses of different study types, we have eliminated potential biases due to different types of effect sizes on the study results. In both prospective or retrospective cohort studies and case-control studies, depression increases the risk of SA, further confirming the reliability of the conclusion. Thirdly, subgroup analyses based on different definitions of spontaneous abortion—including SA before 20 weeks, before 22 weeks, unspecified timing, and RSA—consistently showed a statistically significant association with depression. These results suggest that depression is not only associated with sporadic pregnancy loss across gestational ages but also significantly linked with RSA. However, in the < 24 weeks subgroup, there was no statistical difference in this relationship, possibly due to only one study being included. Fourthly, to eliminate the potential influence of antidepressants on SA, we divided the depressed group into three subgroups: those who did not use antidepressants, those who used antidepressants, and those for whom antidepressant use was not mentioned. The non-depressed subgroup included women who had not been prescribed antidepressants and healthy women. In different subgroups, we found that the above-mentioned positive association still exhibited significant statistical differences, further confirming the close relationship between depression and SA. Fifthly, the positive relationship between depression and SA was found in the subgroups where confounding factors were adjusted. The results after excluding the interference of confounding factors could be considered reliable. However, interestingly, this relationship was not found in the subgroups of studies where confounding factors were not adjusted, possibly due to relatively small sample sizes in these studies. Additionally, the quality of studies that did not adjust for confounding factors was relatively low. Therefore, it can be inferred that the reliability of the results from studies that did not adjust for confounding factors in their subgroups is lower compared to those that did adjust for confounding factors. Sixthly, after conducting subgroup analyses based on different JBI scores, we found that there was a positive association between depression and SA in both high-quality study subgroups and relatively lower-quality study subgroups. This indicates that our research results are not influenced by the quality of the studies included.

This study has several notable strengths, including the large sample size, the inclusion of diverse study designs (cohort and case-control studies) that strengthen causal inference, and broad representation across geographic regions and exposure categories. The rigorous methodology, including comprehensive subgroup and sensitivity analyses, further supports the robustness of the findings. However, several limitations should also be considered: (1) Significant heterogeneity was observed across studies, although subgroup analyses were conducted to explore its sources. (2) Misclassification is possible, as the depression group included women using antidepressants which may have an impact on SA, while the control group may have included some undiagnosed depressed women. (3) The generalizability of findings may be limited by the overrepresentation of European populations and the lack of data from certain geographic regions. (4) In addition, the broad definition of depression used across studies precluded evaluation of specific depression subtypes, and insufficient reporting of antidepressant types prevented analysis by drug class. (5) It should also be noted that the literature search was limited to three databases; PsycInfo and Scopus were not searched due to lack of access, which may have resulted in the omission of relevant studies. (6) The available data did not allow adjustment for several important potential confounders, such as parity, marital status, social support, psychotherapeutic interventions, or psychiatric comorbidities, which may influence the association between depression and spontaneous abortion. Future studies with individual-level data are encouraged to address these factors.

## Conclusion

In summary, this meta-analysis demonstrates a significant association between depression and an increased risk of SA, which remains independent of antidepressant use. These findings underscore the importance of recognizing and addressing depression as a potential risk factor in prenatal care. Future research should further investigate the underlying mechanisms linking depression and SA, examine whether specific subtypes of depression confer differential risks, and explore the relationship between depression and other adverse pregnancy outcomes. In addition, the roles of comorbid conditions and polypharmacy warrant further attention. Well-designed randomized controlled trials are also needed to evaluate the effects of antidepressant medications and psychotherapy on pregnancy outcomes.

## Supplementary Information

Below is the link to the electronic supplementary material.

Supplementary Material 1

Supplementary Material 2

Supplementary Material 3

Supplementary Material 4

## Data Availability

All data generated or analysed during this study are included in this published article [and its supplementary information files].
