# Supplementary material for: Depression with risks for spontaneous abortion: a meta-analysis
Source: BMC Psychol. 2025 Oct 14;13:1148. doi: 10.1186/s40359-025-03484-4 (PMC12522899; doi:10.1186/s40359-025-03484-4)
Supplement: Supplementary file 1 — Supplementary Material 1 [file 40359_2025_3484_MOESM1_ESM.docx]

Additional Table 1 The assessment of study quality

| First author, Year | Whether the inclusion criteria for subjects are clearly defined | Whether to describe the research object and the research site in detail | Whether effective and credible methods are used to assess exposure factors | Whether objective, standardized methods are used to assess health problems | Have confounding factors been identified | Whether measures have been taken to control confounding factors | Whether to use effective and credible methods to measure outcome indicators | Whether the data analysis method is appropriate | JBI Score |
| --- | --- | --- | --- | --- | --- | --- | --- | --- | --- |
| Liang, 2004 | Yes | Yes | Yes | Yes | Yes | Yes | Yes | Yes | 8 |
| Almeida, 2016 | Yes | Yes | Yes | Yes | Yes | Yes | Yes | Yes | 8 |
| Ban, 2016 | Yes | Yes | Yes | Yes | Yes | Yes | Yes | Yes | 8 |
| Yaris, 2005 | Yes | Yes | Yes | Yes | No | No | Yes | Yes | 6 |
| Einarson, 2009 | Yes | Yes | Yes | Yes | Yes | Yes | Yes | Yes | 8 |
| Richardson, 2019 | Yes | Yes | Yes | Yes | Yes | Yes | Yes | Yes | 8 |
| Ankarfeldt, 2021 | Yes | Yes | Yes | Yes | Yes | Yes | Yes | Yes | 8 |
| Sjaarda, 2021 | Yes | Yes | Yes | Yes | Yes | Yes | Yes | Yes | 8 |
| Kitchin, 2022 | Yes | Yes | Yes | Yes | Yes | Yes | Yes | Yes | 8 |
| Wu, 2019 | Yes | Yes | Yes | Yes | Yes | Yes | Yes | Yes | 8 |
| Andersen, 2014 | Yes | Yes | Yes | Yes | Yes | Yes | Yes | Yes | 8 |
| Nakhai-Pour, 2010 | Yes | Yes | Yes | Yes | Yes | Yes | Yes | Yes | 8 |
| Kolding, 2021 | Yes | Yes | Yes | Yes | Yes | Yes | Yes | Yes | 8 |
| Ostenfeld, 2022 | Yes | Yes | Yes | Yes | Yes | Yes | Yes | Yes | 8 |
| Evans-Hoeker, 2018 | Yes | Yes | Yes | Yes | Yes | Yes | Yes | Yes | 8 |
| Johansen, 2014 | Yes | Yes | Yes | Yes | Yes | Yes | Yes | Yes | 8 |
| Kjaersgaard, 2013 | Yes | Yes | Yes | Yes | Yes | Yes | Yes | Yes | 8 |
| Klieger-Grossmann, 2013 | Yes | Yes | Yes | Yes | No | No | Yes | Yes | 6 |
| Wang, 2021 | Yes | Yes | Yes | Yes | Yes | Yes | Yes | Yes | 8 |
| Sivojelezova, 2005 | Yes | Yes | Yes | Yes | No | No | Yes | Yes | 6 |
| Chun-Fai-Chan, 2005 | Yes | Yes | Yes | Yes | No | No | Yes | Yes | 6 |
| Einarson, 2003 | Yes | Yes | Yes | Yes | No | No | Yes | Yes | 6 |
| Einarson, 2001 | Yes | Yes | Yes | Yes | No | No | Yes | Yes | 6 |
| kulin, 1998 | Yes | Yes | Yes | Yes | No | No | Yes | Yes | 6 |
| Goldstein, 1997 | Yes | Yes | Yes | Yes | No | No | Yes | Yes | 6 |
| Koren, 1996 | Yes | Yes | Yes | Yes | No | No | Yes | Yes | 6 |
| Pastuszak, 1993 | Yes | Yes | Yes | Yes | No | No | Yes | Yes | 6 |
| Larson, 2023 | Yes | Yes | Yes | Yes | Yes | Yes | Yes | Yes | 8 |
| Hope, 2022 | Yes | Yes | Yes | Yes | Yes | Yes | Yes | Yes | 8 |
| Diav-Citrin, 2008 | Yes | Yes | Yes | Yes | Yes | Yes | Yes | Yes | 8 |
| Magnus, 2021 | Yes | Yes | Yes | Yes | Yes | Yes | Yes | Yes | 8 |
| Gold, 2007 | Yes | Yes | Yes | Yes | Yes | Yes | Yes | Yes | 8 |

Abbreviation: JBI, Joanna Briggs Institute.
