# Supplementary material for: Depression with risks for spontaneous abortion: a meta-analysis
Source: BMC Psychol. 2025 Oct 14;13:1148. doi: 10.1186/s40359-025-03484-4 (PMC12522899; doi:10.1186/s40359-025-03484-4)
Supplement: Supplementary file 2 — Supplementary Material 2 [file 40359_2025_3484_MOESM2_ESM.docx]

Additional Table 2 Subgroup analyses of the association between depression and spontaneous abortion

| Geographic region | | | | | | | | | | | | | | | | | | | | | | | | | | | | | | | | | | | | | | | | | |
| --- | --- | --- | --- | --- | --- | --- | --- | --- | --- | --- | --- | --- | --- | --- | --- | --- | --- | --- | --- | --- | --- | --- | --- | --- | --- | --- | --- | --- | --- | --- | --- | --- | --- | --- | --- | --- | --- | --- | --- | --- | --- |
| Europe | | | | | North America | | | | | | | | Non-European countries | | | | | Multiple countries | | | | | | | | | Australia | | | | | | | | | | Asia | | | | |
| RR(95% CI) | | P-value | | I^2^ | RR(95% CI) | | | | P-value | | | I^2^ | RR(95% CI) | | | P-value | I^2^ | RR(95% CI) | | | | P-value | | | | I^2^ | RR(95% CI) | | | | P-value | | | | I^2^ | | RR(95% CI) | | | P-value | I^2^ |
| 1.26(1.20,1.34) | | ＜0.001 | | 91% | 1.36(1.19,1.56) | | | | ＜0.001 | | | 71% | 1.91(1.14,3.19) | | | 0.014 | 89% | 1.51(0.79,2.86) | | | | 0.212 | | | | 71% | 1.26(1.16,1.36) | | | | ＜0.001 | | | | 0 | | 1.26(1.04,1.53) | | | 0.019 | NR |
| Study design | | | | | | | | | | | | | | | | | | | | | | | | | | | | | | | | | | | | | | | | | |
| Prospective cohort study | | | | | | | | | | | | | | Retrospective cohort study | | | | | | | | | | | | | | | Case-control study | | | | | | | | | | | | |
| RR(95% CI) | | | P-value | | | | | | | I^2^ | | | | RR(95% CI) | | | P-value | | | | | | | | I^2^ | | | | RR(95% CI) | | | | | P-value | | | | | I^2^ | | |
| 1.35(1.26,1.45) | | | ＜0.001 | | | | | | | 87% | | | | 1.36(1.11,1.67) | | | 0.003 | | | | | | | | 91% | | | | 1.32(1.19,1.46) | | | | | ＜0.001 | | | | | 84% | | |
| Definition of SA | | | | | | | | | | | | | | | | | | | | | | | | | | | | | | | | | | | | | | | | | |
| <20 weeks | | | | | | | | <22 weeks | | | | | | | <24 weeks | | | | | | | | | NR | | | | | | | | | RSA | | | | | | | | |
| RR(95% CI) | P-value | | | I^2^ | | | RR(95% CI) | | | | P-value | | I^2^ | | RR(95% CI) | | P-value | | | | I^2^ | | RR(95% CI) | | | | | P-value | | I^2^ | | RR(95% CI) | | | | | | P-value | | I^2^ | |
| 1.23(1.15,1.31) | ＜0.001 | | | 51% | | | 1.13(1.07,1.19) | | | | ＜0.001 | | 70% | | 1.28(0.85,1.93) | | 0.241 | | | | NR | | 1.49(1.33,1.67) | | | | | ＜0.001 | | 86% | | 1.45(1.35,1.55) | | | | | | ＜0.001 | | 11% | |
| Depressed group | | | | | | | | | | | | | | | | | | | | | | | | | | | | | | | | | | | | | | | | | |
| Depression without mention of antidepressants use | | | | | | | | | | | | | | Depression without taking antidepressants | | | | | | | | | | | | | | | Taking antidepressants | | | | | | | | | | | | |
| RR(95% CI) | | | P-value | | | | | | | I^2^ | | | | RR(95% CI) | | | P-value | | | | | | | | I^2^ | | | | RR(95% CI) | | | | | P-value | | | | | I^2^ | | |
| 1.34(1.19,1.51) | | | ＜0.001 | | | | | | | 80% | | | | 1.16(1.03,1.30) | | | 0.014 | | | | | | | | 60% | | | | 1.38(1.29,1.48) | | | | | ＜0.001 | | | | | 88% | | |
| Non-depressed group | | | | | | | | | | | | | | | | | | | | | | | | | | | | | | | | | | | | | | | | | |
| Healthy | | | | | | | | | | | | | | | | | | | Never been exposed to antidepressants | | | | | | | | | | | | | | | | | | | | | | |
| RR(95% CI) | | | | | P-value | | | | | | | | I^2^ | | | | | RR(95% CI) | | | | | | | | | P-value | | | | | | | | | | I^2^ | | | | |
| 1.35(1.26,1.43) | | | | | ＜0.001 | | | | | | | | 78% | | | | | 1.34(1.23,1.46) | | | | | | | | | ＜0.001 | | | | | | | | | | 90% | | | | |
| Adjustment for confounding factors | | | | | | | | | | | | | | | | | | | | | | | | | | | | | | | | | | | | | | | | | |
| Yes | | | | | | | | | | | | | | | | | | | | No | | | | | | | | | | | | | | | | | | | | | |
| RR(95% CI) | | | | | | P-value | | | | | | | I^2^ | | | | | | | RR(95% CI) | | | | | | | | P-value | | | | | | | | I^2^ | | | | | |
| 1.30(1.24,1.36) | | | | | | ＜0.001 | | | | | | | 86% | | | | | | | 1.49(0.98,2.26) | | | | | | | | 0.061 | | | | | | | | 88% | | | | | |
| JBI Score | | | | | | | | | | | | | | | | | | | | | | | | | | | | | | | | | | | | | | | | | |
| 8 | | | | | | | | | | | | | | | | | | | | 6 | | | | | | | | | | | | | | | | | | | | | |
| RR(95% CI) | | | | | | P-value | | | | | | | I^2^ | | | | | | | RR(95% CI) | | | | | | | | P-value | | | | | | | | I^2^ | | | | | |
| 1.29(1.23,1.35) | | | | | | ＜0.001 | | | | | | | 85% | | | | | | | 1.76(1.02,3.05) | | | | | | | | 0.042 | | | | | | | | 88% | | | | | |

Abbreviation: NR, not reported; SA, spontaneous abortion; RSA, recurrent spontaneous abortion; JBI, the Joanna Briggs Institute; RR, relative risk; CI, confidence interval
